# Reference genome assembly and annotation of two *Bacillus cereus sensu lato* strains from Etosha National Park, Namibia

**DOI:** 10.1128/MRA.00544-23

**Published:** 2023-10-19

**Authors:** Russell J. S. Orr, Ola B. Brynildsrud, Mehdi Abdelli, Vincent Ramisse, Marius Dybwad

**Affiliations:** 1 Total Defence Division, Norwegian Defence Research Establishment FFI, Kjeller, Norway; 2 Division Biologie, DGA Maîtrise NRBC, Vert-le-Petit, France; University of Maryland School of Medicine, Baltimore, Maryland, USA

**Keywords:** genome analysis, *Bacillus cereus*, *Bacillus*

## Abstract

*Bacillus cereus sensu lato* (*s.l*.) poses health and security issues. Here, we report the reference genome assembly of two *Bacillus cereus s.l*. strains, isolated from Etosha National Park, Namibia (FFI_BCgr36 and FFI_BCgr46). These unique genomes open for better understanding of environmental diversity and improvements in detection of threatening species.

## ANNOUNCEMENT

*Bacillus cereus sensu lato* (*s.l*.) includes numerous Gram-positive, spore-forming, and rod-shaped species that are environmentally ubiquitous and pose health and food security issues (1). In particular, *Bacillus anthracis*, the agent of anthrax, the food-borne pathogen *Bacillus cereus sensu stricto,* and the biopesticide *Bacillus thuringiensis* (2).

Two *B. cereus s.l*. strains (FFI_BCgr36 and FFI_BCgr46) were isolated from soil sampled in the vicinity of a *Equus quagga* (plains zebra) carcass (Etosha National Park, Namibia, 2012). Permission was granted from the Ministry of Environment and Tourism of Namibia (permit: 1617/2011). Single-colony isolates were originally cultured from serial dilutions on polymyxin-lysozyme-EDTA-thallous acetate agar plates overnight at 37°C, before storage (3, 4). Isolates were characterized as *B. cereus* using cell and colony morphology, motility, penicillin sensitivity, qPCR, and an MLST scheme (5, 6). Pulsed-field gel electrophoresis characterized plasmid presence and size. Stock was re-cultured overnight at 37°C on tryptic soy agar prior to DNA isolation. DNA was isolated once using Qiagen DNeasy Blood and Tissue kit (Gram-positive protocol). For Illumina, DNA was sheared to 650 bp, and libraries constructed with Nextera XT/DNA prep before MiSeq sequencing. Reads were trimmed using TrimGalore v0.6.10 (7) with –length 80 and –q 30. For Nanopore, libraries were constructed from the same and unsheared genomic DNA with the Rapid Barcoding kit and sequenced on R9.4.1 flow cells, with super-accurate basecalling using Guppy v15.0.0 (8). Reads were trimmed using NanoFilt v2.8.0 (9) with -q 10 -l 5000 and error corrected using FMLRC2 v0.1.7 (10) with cache size 10 and *Kmer* sizes 21, 59, 79, and 127. Hybrid *de novo* assemblies were performed using Unicycler v0.5.0 (11) in “bold” mode allowing circularization of overlapping ends, confirmed with assembly graphs, and rotating assemblies to begin at a consistent starting gene (dnaA). Assemblies were polished with Polypolish v0.5.0 (12) and annotated with PGAP v2022-12-13.build6494 (13). Chromosome and plasmid sequences were compared, at nucleotide level, including closest BLASTn NCBInt hits (January 2023), with nucmer v4.0.0.rc1 (14). Assembly stats were obtained using Bowtie2 v2.5.1 (15) and Minimap2 v2.24-r1122 (16). A chromosome alignment, including closest BLAST hits, was constructed using Parsnp v1.7.4 (17) and phylogenetically inferred with RAxML v8.2.12 (18), employing the GTRCAT model. Topology was the best of 20 heuristic searches and bootstrap from 100 pseudo replicates. The tree was visualized with iTOL (19). Default parameters were used for all software unless otherwise specified.

**Fig 1:**
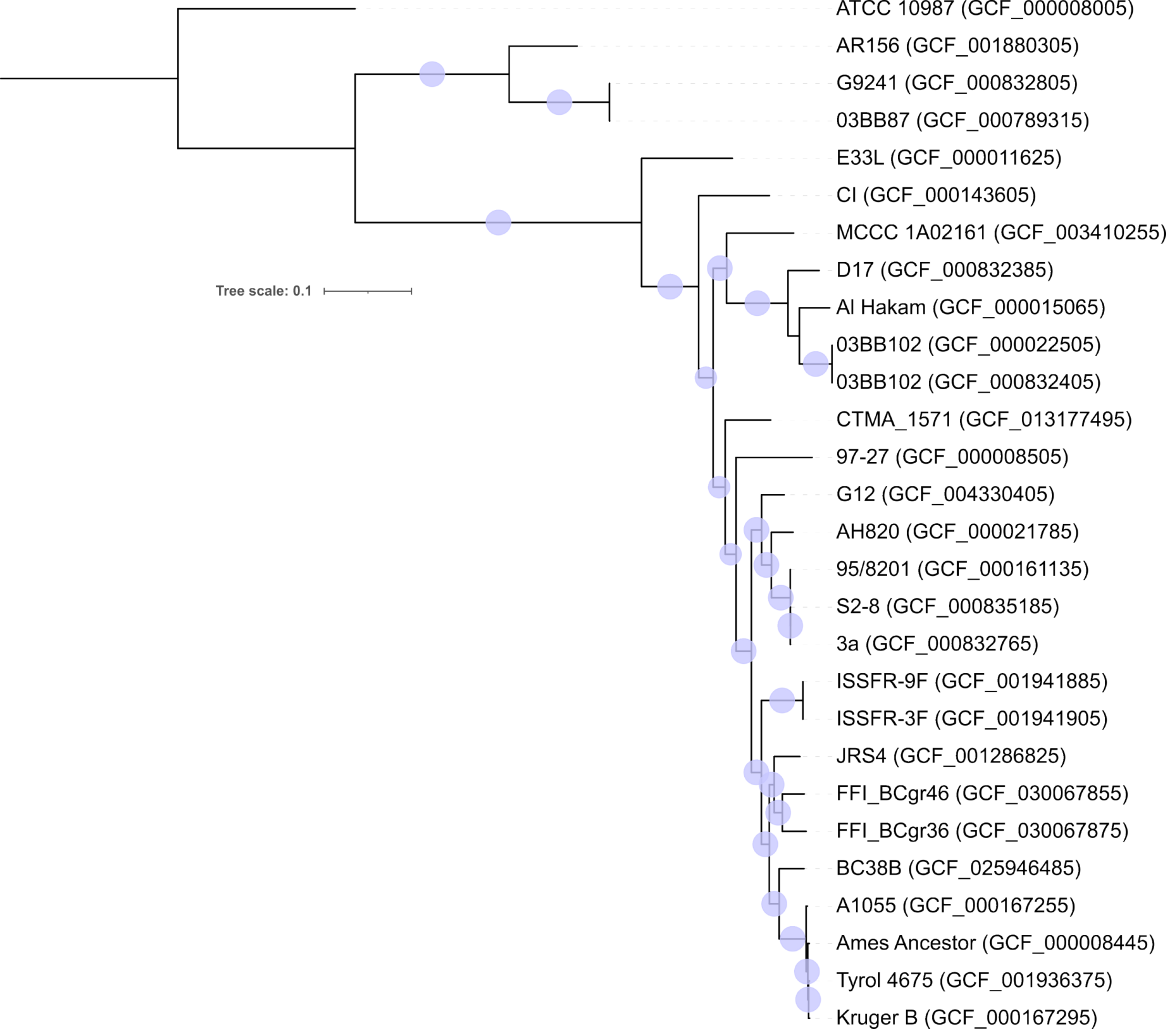
The inferred chromosome phylogeny of multiple *B. cereus s.l*. strains showing the relative position of those presented in this paper. Maximum likelihood phylogeny of 25 *B. cereus s.l*. strains with 190,584 nucleotide characters inferred using RAxML (20 heuristic searches and bootstrap of 100 pseudo replicates). Bootstrap values >90 are shown as blue circles. Assembly accessions for each strain are in brackets.

The two FFI *B. cereus s.l*. strains were assembled to complete circularized chromosome and plasmid sequences (Table 1). FFI_BCgr36 has a 5.35 Mb, and FFI_BCgr46 has a 5.21 Mb chromosome. The annotation (Table 1) and phylogeny (Fig. 1) show strains as highly similar to each other, with the closest public strain being JRS4 *B. cereus* (GCF_001286825.1), isolated from soil, Jeddah (20). *B. cereus* JRS4 has 99.20% BLAST identity, over a 90% query coverage, to both FFI_BCgr36 and FFI_BCgr46 (Fig. 1). For comparison, the FFI strains have a chromosome identity of 99.61% over a 97% query coverage (Fig. 1).

**TABLE 1:** Assembly statistics, sequence data, and genome annotation^*a*^

Assembly	FFI_BCgr36 chromosome	FFI_BCgr36 plasmid 1	FFI_BCgr46 chromosome	FFI_BCgr46 plasmid 1	FFI_BCgr46 plasmid 2
Genbank accession	CP125992	CP125993	CP125989	CP125991	CP125990
Size (bp)	5,352,352	12,239	5,206,453	11,735	579,146
GC %	35.3	35.3	35.4	30.1	32.9
Illumina coverage	116×	771×	122×	489×	137×
ONT coverage	74×	2,753×	56×	1,504×	68×
Comparable depth	1×	6.65×	1×	4×	1×

Sequence data	FFI_BCgr36 genome	FFI_BCgr46 genome
BioSample accession	SAMN35055520	SAMN35055521
SRA accession Illumina	SRX20303850	SRX20303852
Illumina reads (PE)	1,415,409	1,505,971
SRA accession ONT	SRX20303851	SRX20303853
ONT reads	322,909	215,326
ONT N50	12,191	12,311

Annotation	FFI_BCgr36 genome	FFI_BCgr46 genome
Genes (total)	5,558	5,910
CDSs (total)	5,406	5,757
Genes (coding)	5,293	5,594
CDSs (with protein)	5,293	5,594
Genes (RNA)	152	153
rRNAs	14, 14, 14 (5S, 16S, 23S)	14, 14, 14 (5S, 16S, 23S)
Complete rRNAs	14, 14, 14 (5S, 16S, 23S)	14, 14, 14 (5S, 16S, 23S)
tRNAs	105	106
ncRNAs	5	5
Pseudo genes (total)	113	163
CDSs (without protein)	113	163
Pseudo genes (ambiguous residues)	0 of 113	0 of 163
Pseudo genes (frameshifted)	49 of 113	73 of 163
Pseudo genes (incomplete)	70 of 113	93 of 163
Pseudo genes (internal stop)	28 of 113	58 of 163
Pseudo genes (multiple problems)	30 of 113	51 of 163

^*a*^ Statistics from Bowtie2 and Minimap2 for both the chromosome and plasmid of the two *B. cereus s.l*. strains. Annotation for each complete genome from PGAP.

The provided assemblies and annotations permit a better understanding of environmental diversity and improvements in detection of these potentially pathogenic species.

## Data Availability

The complete assemblies and annotations of the two *B. cereus s.l.* strains (FFI_BCgr36 and FFI_BCgr46) have been deposited in Genbank under the following accessions: FFI_BCgr36 chromosome: CP125992; FFI_BCgr36 plasmid: CP125993; FFI_BCgr46 chromosome: CP125989; FFI_BCgr46 small plasmid: CP125990; FFI_BCgr46 large plasmid: CP125991. Associated BioSample accessions including SRAs are SAMN35055520 (FFI_BCgr36) and SAMN35055521 (FFI_BCgr46) within BioProject PRJNA971511.
